# *FLT3*‐ITD DNA and mRNA levels in AML do not correlate with CD7, CD33 and CD123 expression

**DOI:** 10.1111/jcmm.15255

**Published:** 2020-05-27

**Authors:** Dan‐Sebastian Soare, Eugen Radu, Ion Dumitru, Viola Maria Popov, Horia Bumbea, Ana Maria Vlădăreanu

**Affiliations:** ^1^ University Emergency Hospital Bucharest Bucharest Romania; ^2^ Carol Davila University of Medicine and Pharmacy Bucharest Romania; ^3^ Department of Hematology Colentina Clinical Hospital Bucharest Romania

**Keywords:** allelic ratio, AML, *FLT3*‐ITD, MFI, RNA

## Abstract

**Introduction:**

*FLT3* internal tandem duplication (ITD) mutations are found in around 25% of all acute myeloid leukaemia (AML) cases and is associated with shorter disease‐free and overall survival. Previous reports have shown that *FLT3*‐ITD induces a specific phenotype in leukemic blasts, which is characterized by high levels of CD33 and CD123, and that expression of CD33 and CD123 is directly influenced by the DNA *FLT3*‐ITD/wild‐type *FLT3* allelic ratio (AR).

**Methods:**

A total of 42 *FLT3*‐ITD and 104 *FLT3*‐ITD–negative AML patients were analysed. Immunophenotyping data were used to calculate antigen expression levels as the ratio between the geometric mean fluorescence intensities (MFIs) of leukemic blasts and MFIs of negative lymphocyte populations. *FLT3*‐ITD‐DNA and RNA analysis was performed, under the same conditions, by capillary electrophoresis.

**Results:**

Compared with the control group, the *FLT3*‐ITD cohort presented significantly higher CD7, CD33 and CD123 levels. In order to assess the impact of *FLT3*‐ITD abundance on antigen expression, the patients were grouped for each parameter into two cohorts using the following threshold values: (a) 0.5 for the AR, according to current AML guidelines; (b) 0.7 for the *FLT3*‐ITD/*FLT3*‐WT mRNA ratio (RR); and (c) 1.3 for the *FLT3*‐ITD RR/AR ratio. We found higher values of CD33 for RR/AR ≥1.3, and no other statistical differences between CD7, CD33 and CD123 levels of the other *FLT3*‐ITD groups. In terms of correlations between MFI values and *FLT3*‐ITD parameters, we only observed a moderate interdependence between CD33 MFI and the RR/AR ratio, and a weak negative correlation between CD123 MFI and AR.

**Conclusion:**

*FLT3*‐ITD mutations induce a specific antigen profile in AML blasts, and our data do not onfirm previous reports of *FLT3*‐ITD AR influencing both CD33 and CD123 expression.

## 1 INTRODUCTION

Acute myeloid leukaemia (AML) with internal tandem duplication (ITD) insertions within the *FLT3* gene represents around 25% of all AML cases, although *FLT3*‐ITD AML is not a distinct entity according to the 2016 revision of the World Health Organization classification.^3^ These patients present a worse prognosis than patients without the mutation, with a shorter disease‐free survival and overall survival.^1^, ^2^

At diagnosis, *FLT3*‐ITD AML patients present high white blood cell counts, with a high percentage of bone marrow and circulating blasts. Also, *FLT3*‐ITD AML cells have a characteristic high CD33 and CD123 expression,^4^, ^5^, ^6^ which was shown to be directly proportional to the DNA *FLT3*‐ITD/*FLT3*‐WT allelic ratio (AR).^4^

In this study, we investigated the quantitative expression of cell surface markers in *FLT3*‐ITD AML from our local patient population, and evaluated the impact of both DNA and mRNA *FLT3*‐ITD/*FLT3*‐WT ratios on antigen expression levels.

## 2 MATERIALS AND METHODS

### 2.1 Patients

A total of 42 AML patients with the *FLT3*‐ITD mutation, diagnosed between March 2016 and June 2019, and a control group of 104 *FLT3*‐ITD–negative AML patients were included in this study. All patients gave written informed consent, and the study was conducted in accordance with the Declaration of Helsinki and Good Clinical Practice guidelines. Patient demographic details are presented in Appendix S1 and in Table S1.

### 2.2 Immunophenotyping

All primary diagnostic samples, bone marrow aspirate or peripheral blood samples, were processed and analysed according to EuroFlow recommendations.^7^, ^8^ The following antigens were quantitatively analysed to determine the specific *FLT3*‐ITD blast expression profile: CD4, CD7, CD9, CD13, CD14, CD33, CD34, CD56, CD64, CD71, CD117 and CD123. Antigen expression levels were calculated as the geometric mean fluorescence index (MFI) ratios between a blast population and a lymphocyte population negative for the respective marker, as previously described^4^⁠ (see also Appendix S1 and Figure S1).

### 2.3 *FLT3*‐ITD DNA and mRNA analyses

We performed *FLT3*‐ITD DNA and mRNA qualitative and quantitative analyses by capillary electrophoresis, as previously described.^9^, ^10^ Three *FLT3*‐ITD parameters were calculated: the *FLT3*‐ITD‐to‐*FLT3*‐WT (a) allelic ratio (AR), (b) mRNA expression ratio (RR) and the (c) relative abundance of *FLT3*‐ITD mRNA to DNA calculated as the RR/AR ratio. Details regarding the assay and *FLT3*‐ITD parameters are presented in Appendix S1—methods, results and Figure S2.

### 2.4 Statistical analyses

See details in Appendix S1.

## 3 RESULTS

### 3.1 *FLT3*‐ITD–specific antigen expression profile

**FIGURE 1:**
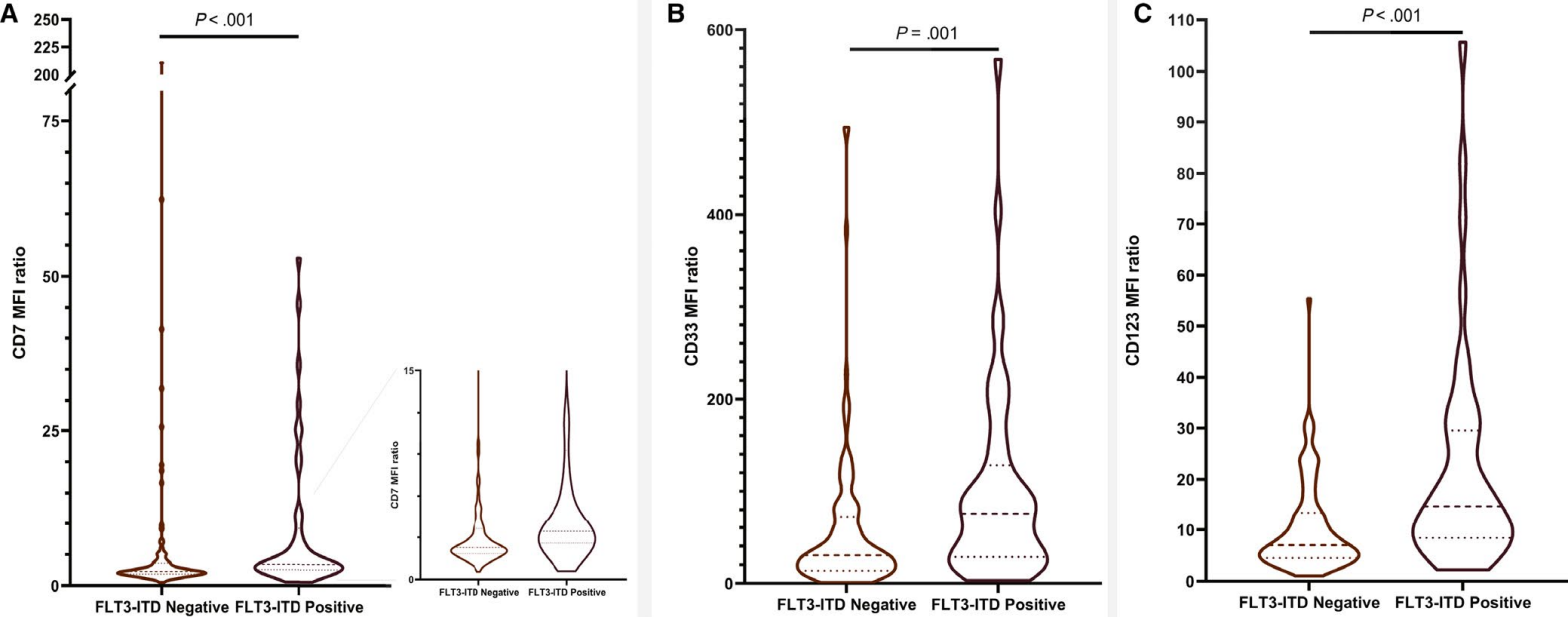
MFI ratios for (A) CD7 (inset: a close‐up of the plot), (B) CD33 and (C) CD123 in *FLT3*‐ITD–positive *vs FLT3*‐ITD–negative AML patients. AML, acute myeloid leukaemia; ITD, internal tandem duplication; MFI, mean fluorescence index

*FlT3*‐ITD–positive cases presented a significantly higher CD7, CD33 and CD123 expression when compared to the *FLT3*‐ITD–negative control group (Figure 1) (Mann‐Whitney *U* test; see also Table S2).

### 3.2 *FLT3*‐ITD quantitative parameters in relation to CD7, CD33 and CD123 expression profiles

**FIGURE 2:**
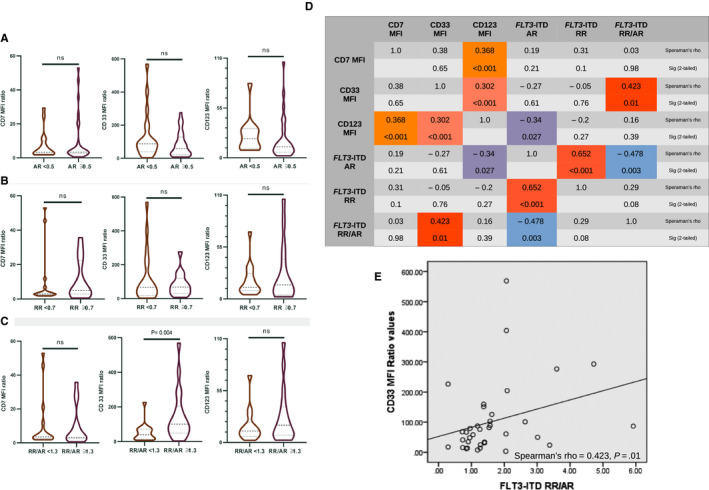
Detailed analysis of CD7, CD33 and CD123 MFI values according to *FLT3*‐ITD quantitative parameters. Violin plots of antigen expression levels according to the *FLT3*‐ITD (A) AR, (B) RR and (C) RR/AR ratio. D, Results of correlation analysis between CD7, CD33 and CD123 and AR, RR and RR/AR ratio. E, Correlation between CD33 MFI ratio and RR/AR ratio. ITD, internal tandem duplication; MFI, mean fluorescence index; AR, *FLT3*‐ITD/*FLT3*‐WT DNA allelic ratio; RR, *FLT3*‐ITD/*FLT3*‐WT mRNA ratio; RR/AR, relative abundance of *FLT3*‐ITD mRNA to DNA

To evaluate the association of *FLT3*‐ITD quantitative parameters and CD7, CD33 and CD123 expression, *FLT3*‐ITD–positive patients were grouped for each parameter into two cohorts using the following threshold values: (a) 0.5 for *FLT3*‐ITD AR (the genetic prognostic cut‐off according to the ELN 2017 guidelines^1^), (b) 0.7 for the RR (median value) and (c) 1.3 for the RR/AR ratio (median value). Additional details regarding *FLT3*‐ITD mutation parameters can be found in Appendix S1—results section, Table S3. The only difference observed was between the RR/AR ratio groups, with the high RR/AR group (≥1.3) presenting higher values of CD33 than the low RR/AR group (Figure 2C). There were no other statistical differences between CD7, CD33 and CD123 levels of the other *FLT3*‐ITD groups (Mann‐Whitney *U* test; Figure 2).

We also tested the bivariate correlation between CD7, CD33 and CD123 expression and the three *FLT3*‐ITD quantitative parameters. There was a moderate correlation between CD33 MFI values and RR/AR ratio values (Spearman's rho = 0.423, *P* = .01) (Figure 2D,E). There was also a weak negative correlation between CD123 MFI values and AR (Spearman's rho = −0.34, *P* = .027) (Figure S3). There were no other correlations between MFI values and *FLT3*‐ITD parameters.

To further investigate the relation between CD33 MFI values and the *FLT3*‐ITD RR/AR ratio, we evaluated the repartition of *NPM1* mutations within the two RR/AR groups (cut‐off value of 1.3), given that CD33 values are also elevated in *NPM1*‐mutated AMLs.^4^, ^6^, ^11^ The high RR/AR (≥1.3) group presented statistically more *NPM1* mutant cases (11 positive/15 negative cases) than the low RR/AR (<1.3) group (6 positive/16 negative) (Pearson's chi‐square = 4.041, *P* = .045).

Given that patients with high RR/AR ratios had a higher percentage of *NPM1* mutations, we evaluated the relationship between RR/AR ratio values and *NPM1* status by performing a receiver operating characteristic (ROC) curve (Figure S4). There was no significant correlation between RR/AR values and *NPM1* status (area under the curve = 0.676, 95% CI: 0.484‐0.869, *P* = .95).

## 4 DISCUSSION

In our study population, the presence of *FLT3*‐ITD correlated with a specific antigen profile, characterized by higher expression of CD7, CD33 and CD123 when compared to the *FLT3*‐ITD–negative control population. Increased CD33 and CD123 expression has been previously reported in *FLT3*‐ITD leukaemic cells.^4^, ^5^, ^6^ Some previous reports^12^, ^13^ described increased CD7 levels, similar to our findings; however, in the study by Haubner et al^6^ neither myeloblasts nor leukaemic stem cells had higher MFI values for CD7, suggesting a difference between patient cohorts.

A significantly higher expression of CD33 was seen in the high RR/AR group (≥1.3) when compared to the low RR/AR group (<1.3) (Figure 2C). When we further investigated the association between *FLT3*‐ITD quantitative parameters of leukaemic cells and antigen expression, we also observed a moderate correlation between CD33 and RR/AR values (Figure 2D,E). However, when we evaluated the presence of *NPM1* insertion mutations, which are constantly associated with high CD33 expression,^4^, ^6^, ^11^ we observed that *FLT3*‐ITD–positive AMLs with higher RR/AR ratios presented a higher prevalence of *NPM1* mutations. This could explain both the higher values of CD33 MFI ratios in the high RR/AR group and the bivariate correlation between the two parameters.

In contrast to a previous report of an association between higher AR values and higher CD33 and CD123 MFI values,^4^ we could not confirm this correlation in our study. This could represent a difference between the two patient cohorts, mainly suggested by the fact that we did not observe any positive correlations between CD33 or CD123 and AR values when using two different statistical tests (non‐parametric test and bivariate correlation). Moreover, we observed a weak, yet statistically significant, negative correlation between AR values and CD123 MFI values (Figure 2D, Figure S3). In a recent report by Sung et al,^14^ CD123 signalling (IL‐3R) was found to rescue *FLT3*‐ITD primary leukaemia cells after exposure to *FLT3* tyrosine kinase inhibitors, promoting cell survival but not proliferation. Also, in a previous paper by Choudhary et al,^15^ when using a cellular viability assay with *Flt3*‐ITD–overexpressing 32D cells, the addition of IL‐3 ligand showed to provide antiapoptotic signalling through STAT5a/b activation. One of the main hallmarks of *FLT3*‐ITD signalling is the aberrant activation of the STAT5 transcription factor.^2^ Finally, this could indicate that in our previously untreated *FLT3*‐ITD patient population, cases with a lower AR are more dependent of CD123 signalling, with further studies being required to evaluate this hypothesis.

In our patient population, *FLT3*‐ITD mutations were associated with a specific antigen expression profile consisting of high MFI values for CD7, CD33 and CD123. However, antigen expression levels are not obviously influenced by *FLT3*‐ITD DNA and mRNA quantitative parameters. These results suggest that AML patient populations can be further stratified based on differences in phenotype, as well as subtle differences in the genotype of leukaemic cells.

## CONFLICT OF INTEREST

There are no conflicts of interests to declare.

## AUTHOR CONTRIBUTIONS

DSS and ID performed the research. DSS, ER, VP, HB and AMV designed the research study. ER and HB contributed essential tools. DSS and ER analysed the data. DSS and ER wrote the manuscript.

## Supporting information

Appendix S1

## Data Availability

The data that support the findings of this study are available from the corresponding author upon reasonable request.
