# Supplementary material for: FLT3‐ITD DNA and mRNA levels in AML do not correlate with CD7, CD33 and CD123 expression
Source: J Cell Mol Med. 2020 May 27;24(13):7675–9. doi: 10.1111/jcmm.15255 (PMC7339186; doi:10.1111/jcmm.15255)

**Materials and Methods**

**Patients**

Forty-two AML patients were diagnosed with *FLT3*-ITD after DNA analyses. Of these, 32 patients also presented good quality mRNA samples. Sample UPN-8334, presented a *FLT3*-ITD mutation only on the DNA sample, whereas on the mRNA sample only the *FLT3*-WT amplicon was detected. In the end 31 mRNA samples presented a *FLT3*-ITD mutation and were further analyzed.

A control group formed of 104 FLT3-ITD negative patients, was matched for sex, age, FAB subtype, cytogenetics, and NPM1 mutation status with the *FLT3*-ITD positive patients – Table S1.

**Immunophenotyping**

All samples were analyzed according to the EuroFlow protocols: sample preparation, instrument setting and calibration [1] ⁠; and antibody panels, staining procedure and data acquisition [2] ⁠. Samples were analyzed on a FACSCanto II cytometer (BD Biosciences). A total of 20 000 events per sample were recorded. Measurements and analyses of acquired data was performed using FlowJo software X (Tree Star, Ashland, OR, USA). The gating strategy was based on the CD45/side scatter (SSC) gate. Leukemic blasts cell populations were defined as CD45-mid & SSC-low. To verify the gating strategy the selected populations were backdated according to CD117-, CD34-, and HLA-DR- expression (Figure S1 – A).

The selection of specific antigens to test was based on a screening analyses of local immunophenotyping reports (data not shown), and on previously published reports regarding antigen expression in *FLT3*-ITD/*NPM1*-MUT AML[3–11] ⁠.The geometric mean fluorescence intensities (MFIs) of CD4, CD7, CD9, CD13, CD14, CD33, CD34, CD56, CD64, CD71, CD117, and CD123 were determined for the leukemic blasts populations and control lymphocyte populations (CD45-bright & SSC-low), negative for the respective markers (Figure S1 – B, C). Further the expression of specific antigens was determined as the ration between the specific blasts MFI and the control lymphocyte MFI, as previously described [12]⁠.

***FLT3*-ITD DNA and mRNA analyses**

Genomic DNA was isolated from mononuclear cells obtained from bone marrow aspirate where available or from peripheral blood samples using Biocoll separating solution (Biochrom GmbH, Berlin, Germany). DNA extraction was performed using GENXTRACT Blood DNA Extraction System (ViennaLab Diagnostics GmbH, Vienna, Austria) according to the manufacturer’s recommendations. Total RNA was isolated from bone marrow aspirate or peripheral blood samples preserved in PAXgene Blood RNA Tube and extracted using the QIAsymphony PAXgene Blood RNA Kit (PreAnalytiX GmbH, Hombrechtikon, Switzerland) on the QIAsymphony automated system (Qiagen, Hilden, Germany) as per the manufacturers’ instructions. Revers transcription was performed using the GoScript RT Mix kit (Promega, USA) as per the manufacturer’s instructions.

For *FLT3*-ITD analyses a hot-start polymerase chain reaction (PCR) amplification of genomic DNA and total RNA was performed with florescent-labeled primers. The FLT3 primers used covered the region spanning exons 14 and 15 of the *FLT3* gene, as previously described [13, 14]. The PCR conditions used were – initial denaturation 10 minutes at 95ºC; followed by 25 cycle each 45 seconds at 95ºC, 45 seconds at 62ºC, 45 seconds at 72ºC; with a final extension 6 minutes at 72ºC. The gDNA *FLT3*-WT amplicon length was 330 bp and the cDNA *FLT3*-WT was 239 bp. *FLT3*-ITD mutations were considered present if amplicons longer than the *FLT3*-WT products were detected, for both the DNA and mRNA analyses by capillary electrophoresis and fragment analyses on a CEQ8000 DNA Genetic Analysis System (Beckman Coulter UK, High Wycombe, United Kingdom). Genomic DNA contamination of cDNA samples was considered when on the fragment analyses there were present amplicons corresponding in length to the gDNA *FLT3*-WT and/or the gDNA *FLT3*-ITD products. Genomic DNA contamination was removed by using the DNase Max Kit (Qiagen, Hilden, Germany). The threshold for positive mutations was at 0.5% of the WT amplicon area under the peak (AUP) for either gDNA or mRNA samples. If a patient presented an abnormal AUP of 0.5%, the PCR reaction was repeated and the PCR product was re-analyzed on the CEQ8000 system, while applying the same positivity threshold.

The *FLT3*-ITD DNA allelic ratio (AR) was calculated as the AUP*^FLT3^*^-ITD^/AUP*^FLT3^*^-WT^. In patients with multiple *FLT3*-ITD populations (≥2 *FLT3*-ITD amplicons) the allelic ration was calculated by summing the AUP for each *FLT3*-ITD population and then dividing by the AUP*^FLT3^*^-WT^. The quantify the level *FLT3*-ITD mRNA, an expression mRNA ratio (RR) was calculated as described above for the allelic ratio. To test the influence of the relative *FLT3*-ITD expression on the antigen expression pattern, a third parameter was calculated as the ratio between the RNA expression ration and the allelic ratio. This parameter determined was the relative abundance of *FLT3*-ITD mRNA to DNA, calculated as the RR/AR ratio. An exemplary patient DNA and mRNA analyses are presented in Figure S2.

**Statistical analyses**

All data sets presented non-normal distributions and the Mann-Whitney U test was used to compare MFI values between different populations. Spearman’s correlation was used to evaluate the relationship between different continuous variables. Categorical variables were analyzed using Pearson’s chi-square or Fischer’s exact test where appropriate. P values of <0.05 were considered to be statistically significant with only the 2-sided P values being used. Statistical analyses were performed using SPSS software (version 23) and GraphPad Prism 8.

**Results**

***FLT3*-ITD parameters & patient grouping according to quantitative parameters**

*FLT3*-ITD parameters for both DNA and mRNA samples are presented in Table S3. Given that we did not evaluate the impact of ITD insert length on antigen expression, only the first ITD populations are presented in Table S3.

For the analyses of the impact of the AR on MFI values the patients were separated into 2 groups according to the 2017 ELN genetic risk stratification [15]⁠ into an AR <0.5 group (n= 16) and an AR ≥0.5 group (n= 26). For the analyses of the RR the patients were separated into 2 groups according to the median RR (= 0.72): a RR <0.7 group (n= 16) and a RR ≥0.7 group (n= 15). For the analyses of the relative abundance of *FLT3*-ITD mRNA to DNA (RR/AR) the patients were separated into 2 groups according to the median RR/AR (= 1.27): a RR <1.3 group (n= 16) and a RR ≥1.3 group (n= 15).

**References**

[1] **Kalina T, Flores-Montero J, Van Der Velden VHJ, et al.** EuroFlow standardization of flow cytometer instrument settings and immunophenotyping protocols. *Leukemia* 2012; 26; 1986–2010.

[2] **Van Dongen JJM, Lhermitte L, Böttcher S, et al.** EuroFlow antibody panels for standardized n-dimensional flow cytometric immunophenotyping of normal, reactive and malignant leukocytes. *Leukemia* 2012; 26; 1908–75.

[3] **Mori Y, Yoshimoto G, Kumano T, et al.** Distinctive expression of myelomonocytic markers and down-regulation of CD34 in acute myelogenous leukaemia with FLT3 tandem duplication and nucleophosmin mutation. *Eur. J. Haematol.* 2007; 79; 17–24.

[4] **Rausei-Mills V, Chang KL, Gaal KK, et al.** Aberrant expression of CD7 in myeloblasts is highly associated with de novo acute myeloid leukemias with FLT3/ITD mutation. *Am. J. Clin. Pathol.* 2008; 129; 624–9.

[5] **Riccioni R, Diverio D, Riti V, et al.** Interleukin (IL)-3/granulocyte macrophage-colony stimulating factor/IL-5 receptor alpha and beta chains are preferentially expressed in acute myeloid leukaemias with mutated FMS-related tyrosine kinase 3 receptor. *Br. J. Haematol.* 2009; 144; 376–87.

[6] **Chauhan PS, Bhushan B, Mishra AK, et al.** Mutation of FLT3 gene in acute myeloid leukemia with normal cytogenetics and its association with clinical and immunophenotypic features. *Med. Oncol.* 2011; 28; 544–51.

[7] **Dalal BI, Mansoor S, Manna M, et al.** Detection of CD34, TdT, CD56, CD2, CD4, and CD14 by flow cytometry is associated with NPM1 and FLT3 mutation status in cytogenetically normal acute myeloid leukemia. *Clin. Lymphoma, Myeloma Leuk.* 2012; 12; 274–9.

[8] **Rollins-Raval M, Pillai R, Warita K, et al.** CD123 immunohistochemical expression in acute myeloid leukemia is associated with underlying FLT3-ITD and NPM1 mutations. *Appl. Immunohistochem. Mol. Morphol.* 2013; 21; 212–7.

[9] **Chauhan PS, Ihsan R, Singh LC, et al.** Mutation of NPM1 and FLT3 genes in acute myeloid leukemia and their association with clinical and immunophenotypic features. *Dis. Markers* 2013; 35; 581–8.

[10] **Baqai J, Crisan D**. Correlation of FLT3 mutations with expression of CD7 in acute myeloid leukemia. *Appl. Immunohistochem. Mol. Morphol.* 2015; 23; 104–8.

[11] **Al-Mawali A, Gillis D, Lewis I**. Immunoprofiling of leukemic stem cells CD34+/CD38-/CD123+ delineate FLT3/ITD-positive clones. *J. Hematol. Oncol.* 2016; 9.

[12] **Ehninger A, Kramer M, Röllig C, et al.** Distribution and levels of cell surface expression of CD33 and CD123 in acute myeloid leukemia. *Blood Cancer J.* 2014; 4; e218–e218.

[13] **Thiede C, Steudel C, Mohr B, et al.** Analysis of FLT3-activating mutations in 979 patients with acute myelogenous leukemia: Association with FAB subtypes and identification of subgroups with poor prognosis. *Blood* 2002; 99; 4326–35.

[14] **Gale RE, Green C, Allen C, et al.** The impact of FLT3 internal tandem duplication mutant level, number, size, and interaction with NPM1 mutations in a large cohort of young adult patients with acute myeloid leukemia. *Blood* 2008; 111; 2776–84.

[15] **Döhner H, Estey E, Grimwade D, et al.** Diagnosis and management of AML in adults: 2017 ELN recommendations from an international expert panel. *Blood* 2017; 129; 424–47.

| **Supporting Table 1. Clinical characteristics of *FLT3*-ITD patients compared with control group (total n= 146)** | | | |
| --- | --- | --- | --- |
| **Feature** | ***FLT3*-ITD positive**  **(n=42)** | ***FLT3*-ITD negative**  **(n= 104)** |  |
| Sex M/F, n | 16/26 | 54/50 | P= NS^†^ |
| Age, y | Median 64.5  Range 24 – 80 | Median 66.5  Range 24 – 81 | P= NS* |
| FAB subtypes included, n |  |  | P= NS** |
| M1 | 24 | 58 |  |
| M2 | 2 | 8 |  |
| M4 | 10 | 21 |  |
| M5 | 6 | 17 |  |
| Cytogenetics, n |  |  | P= NS** |
| Favorable | 1 | 5 |  |
| Normal | 21 | 46 |  |
| Intermediate | 3 | 9 |  |
| Poor, of which w. complex karyotype | 3, 1 | 10, 6 |  |
| Insufficient material | 2 | 9 |  |
| Unknown | 12 | 25 |  |
| *NPM1* mutations, n | 21 (50%) | 34 (39.2%) | P= 0.6^†^ |
| *Mann-Whitney U-test  ** Fischer’s exact test  ^†^Pearson’s Chi-Square | | | |

| **Supporting Table 2. Antigen MFI values of blast cells in *FLT3*-ITD AML patients compared with a *FLT3*-ITD negative control group** | | | |
| --- | --- | --- | --- |
| Antigen | ***FLT3*-ITD (n=42)** | **Control (n= 104)** |  |
| CD4 | Median 8.52  Range 0.07 – 111.43 | Median 8.61  Range 0.2 – 154.05 | P= NS |
| CD7 | Median 3.39  Range 0.59 – 52.98 | Median 2.28  Range 0.54 – 211.03 | **P <0.001** |
| CD9 | Median 7.06  Range 1.96 – 97.5 | Median 7.53  Range 0.24 – 124.54 | P= NS |
| CD13 | Median 21.23  Range 2.5 – 228.95 | Median 20.63  Range 1.3 – 357.91 | P= NS |
| CD14 | Median 6.27  Range 0.3 – 165.94 | Median 5.53  Range 0.07 – 455.26 | P= NS |
| CD33 | Median 75.92  Range 3.18 – 568.59 | Median 30.2  Range 1.04 – 494.21 | **P= 0.001** |
| CD34 | Median 7.81  Range 0.66 – 962.74 | Median 12.44  Range 0.73 – 637.09 | P= NS |
| CD56 | Median 2.89  Range 0.47 – 18.51 | Median 3.03  Range 0.01 – 381.48 | *P= NS* |
| CD64 | Median 9.92  Range 1.03 – 194 | Median 15.44  Range 0.61 – 913.38 | P= NS |
| CD71 | Median 6.98  Range 0.87 – 51.84 | Median 6.41  Range 0.82 – 56.38 | P= NS |
| CD117 | Median 37.6  Range 0.82 – 364 | Median 29.27  Range 1.08 – 952.6 | P= NS |
| CD123 | Median 14.74  Range 2.24 – 105.8 | Median 7.05  Range 1.07 – 55.49 | **P <0.001** |
| Mann-Whitney U test | | | |

| **Supporting Table 3. *FLT3*-ITD mutation characteristics** | | |
| --- | --- | --- |
| **Feature** | **DNA *FLT3*-ITD (n= 42)** | **mRNA *FLT3*-ITD (n= 31)** |
| Mutation length – first population, bp | Median 43.5  Range 6 – 126 | Median 42  Range 6 – 93 |
| *FLT3*-ITD/-WT ratio | Median 0.57  Range 0.03 – 2.29 | Median 0.72  Range 0.05 – 5.46 |
| *FLT3*-ITD relative expression (RR/AR) |  | Median 1.28  Range 0.29 – 5.92 |
| AR (allelic ratio), RR (mRNA ratio) | | |


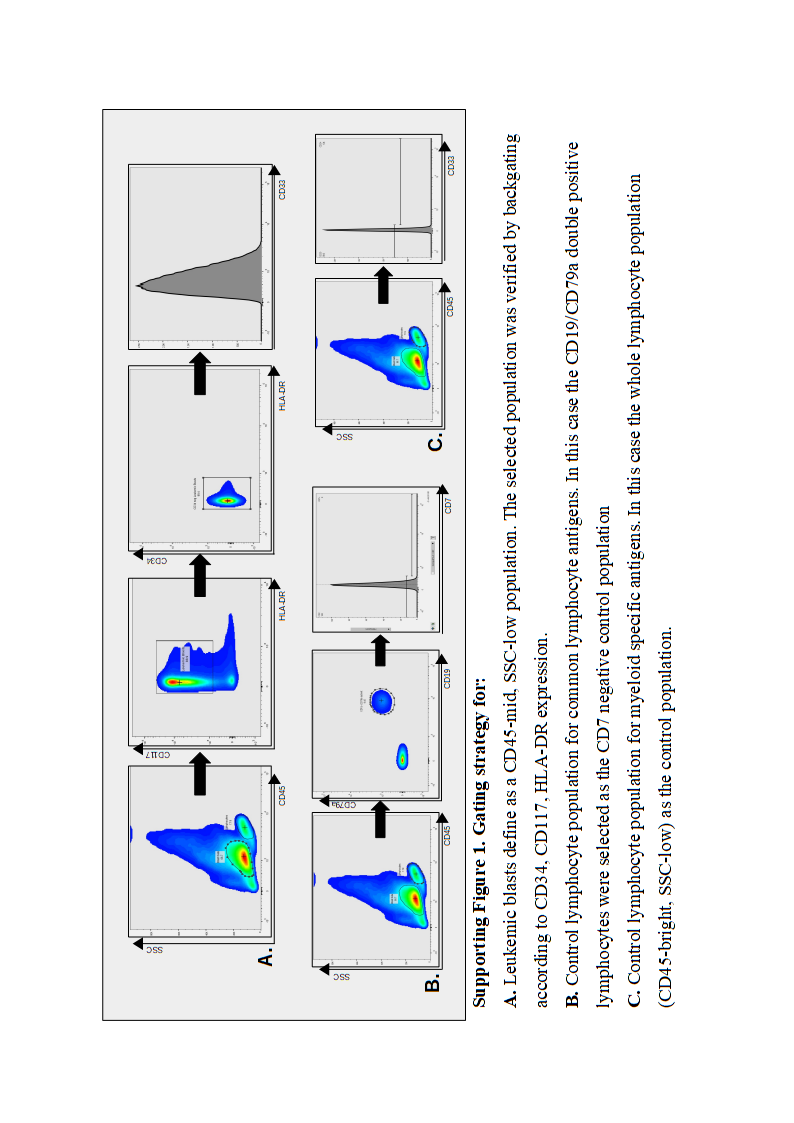


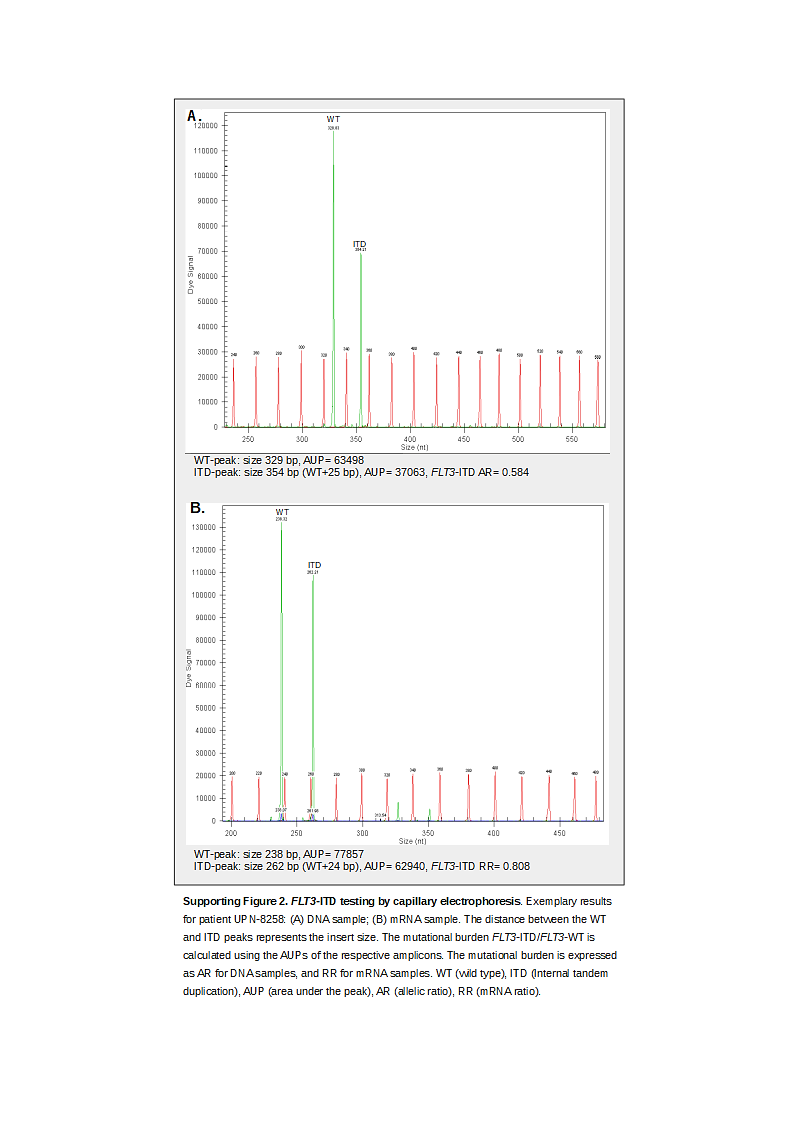


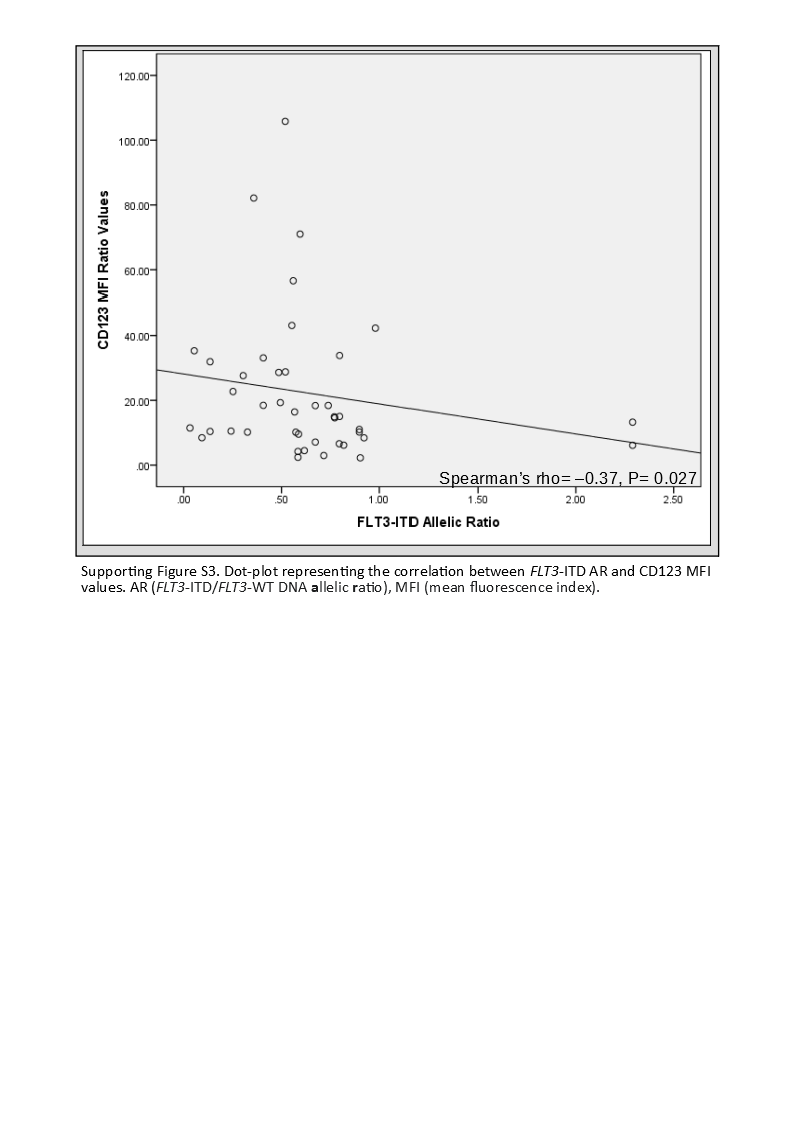


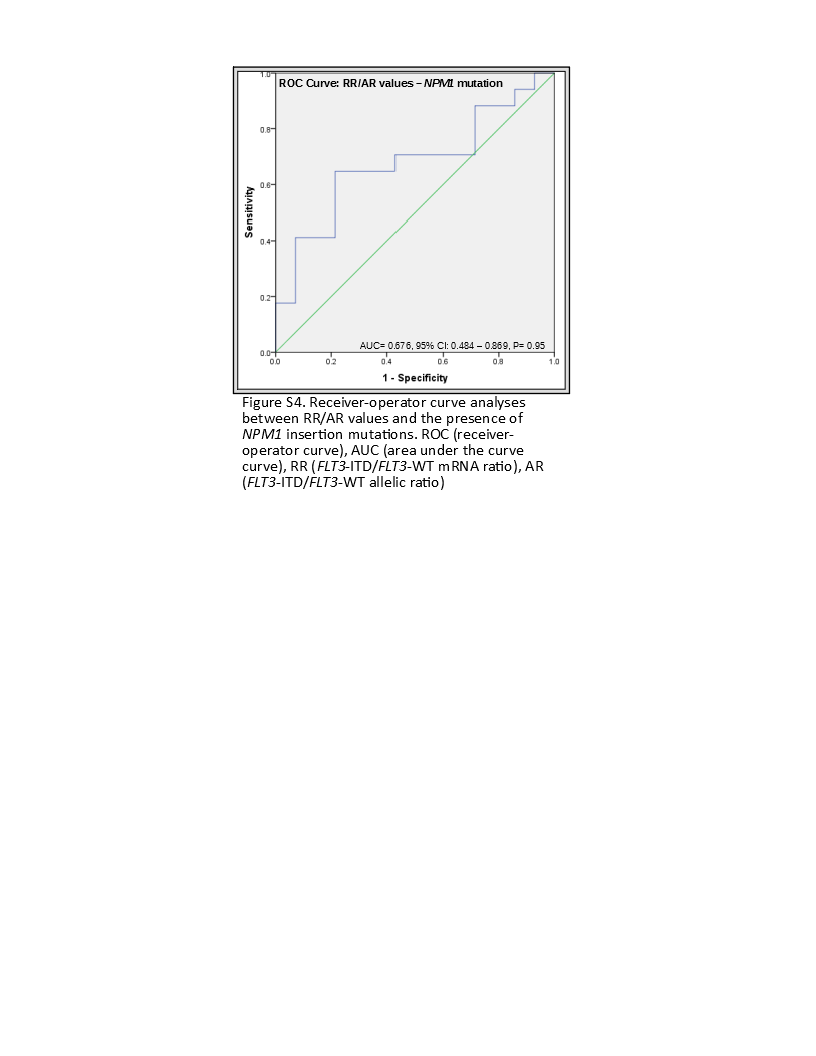

Supplement: Supplementary file 1 — Appendix S1 [file JCMM-24-7675-s001.docx]
